# Ichthyol: Its Internal and External Uses

**Published:** 1888-09

**Authors:** 


Ichthyol: Its Internal and External Uses.—Prof. Nussbaum (*Wein Med. Woch.*) has obtained remarkable effects with ichthyol. Cures are cited in divers forms of neuralgia, bone pains, articular pains, and muscular and gouty troubles. The medicament was given in pills containing one-sixth grain. Patients commenced by taking two pills twice daily, the amount being rapidly increased to two pills five times a day. Two pills, twelve times daily, may be taken if necessary. The drug is withdrawn as soon as the effect is obtained. In case of relapse, the medicament is given again in such doses as have proved active in the preceding treatment. Ichthyol in high doses is not injurious; Nussbaum often gave seventy-seven grains *per diem*. For external use, Fisher recommends the following formulæ: Ointment for articular and other rheumatic pains, gouty pains, psoriasis, prurigo and burns: Ichthyol, one; lanoline, nine. Ointment for eczema: Ichthyol, one; ung. diachylon, twenty.—*Monit. Thérap.*, *March 5th.*

